# Exploring the impact of breast cancer support groups on survivorship and treatment decision-making in eastern Ethiopia: a qualitative study

**DOI:** 10.1007/s00520-025-09475-w

**Published:** 2025-04-26

**Authors:** Nahom G. Belete, Meera Bhakta, Tara Wilfong, Mahlet Shewangizaw, Edilawit Abebaw Abera, Yehenaw Tenaw, Michael Shawel, Habtamu Seife, Biruk Habtamu, Nahom Wondwossen, Elizabeth A. Wood

**Affiliations:** 1 Hiwot Fana Specialized University Hospital, Harar, Ethiopia; 2 College of Science, University of Notre Dame, Notre Dame, IN USA; 3 School of Public Health, College of Health and Medical Sciences, Haramaya University, Harar, Ethiopia; 4 Eck Institute for Global Health, University of Notre Dame, Notre Dame, IN USA

**Keywords:** Support groups, Survivorship, Breast cancer, Ethiopia, Qualitative

## Abstract

**Purpose:**

This study explores the psychosocial impact of breast cancer (BC) support groups on survivorship and treatment decision-making among women in Harar, Ethiopia. It examines the influence of cultural, social, and economic factors on treatment decisions and assesses the effectiveness of support groups in addressing these challenges.

**Methods:**

A community-based case study used semi-structured, in-depth interviews and focus group discussions. Participants included women attending BC support groups and key informants, including healthcare providers, caregivers, and spiritual leaders. Data were collected at the Hiwot Fana Cancer Treatment Center and were analyzed thematically using an inductive approach to identify key themes.

**Results:**

The study highlighted significant barriers to timely BC treatment, including cultural stigma, financial constraints, reliance on alternative medicine, and limited healthcare infrastructure. Support groups were pivotal in improving emotional and psychological well-being, fostering a sense of community, and influencing treatment decision-making. Participants reported increased awareness, reduced stigma, and enhanced community advocacy.

**Conclusions:**

BC support groups in Harar address critical gaps in cancer care by providing psychosocial support and mitigating barriers to treatment. These groups also serve as platforms for community education and advocacy, promoting early detection and modern treatment practices.

**Implications for cancer survivors:**

Support groups empower BC survivors by improving emotional resilience, facilitating informed treatment decisions, and fostering a sense of belonging. They also help reduce stigma and build supportive community networks essential for long-term survivorship in low-resource settings. Expanding access to such groups could significantly enhance cancer care outcomes in Ethiopia and similar contexts.

**Supplementary Information:**

The online version contains supplementary material available at 10.1007/s00520-025-09475-w.

## Introduction

Cancer is one of the leading causes of death globally, with an estimated 10 million deaths in 2020 [1]. In Africa, both the incidence and mortality rates of cancer are escalating, with 847,000 new cases and 591,000 deaths reported in 2012 [2]. The United Nations estimates that Africa’s population will grow by 60% between 2010 and 2030, with a 90% increase in individuals aged 60 and older, the age group most susceptible to cancer [2]. Breast cancer (BC) continues to be the second leading cause of death among women worldwide, with rising incidence rates, particularly in developing countries where most cases are diagnosed at advanced stages [3]. BC poses a significant threat globally, particularly impacting women and serving as a public health challenge in sub-Saharan Africa (SSA) [4]. Data quality and availability on BC mortality and incidence from low- and middle-income countries in the Global Cancer Observatory (GLOBOCAN) project are generally poor [1]. This issue is exacerbated in SSA, where there is a notable lack of incidence and mortality statistics [5]. The paucity of epidemiological or clinical data around BC in SSA, specifically Ethiopia, obscures the actual burden of disease, with only 20 (43.4%) of the 46 World Health Organization (WHO) member states in SSA maintaining active cancer registries [6]. Consequently, BC may be neglected in SSA as governments prioritize other health issues, particularly communicable diseases. Limited healthcare funding in SSA countries, reflected in low GDP expenditure on healthcare, contributes to inadequate health infrastructure, staffing, and awareness, further exacerbating the lack of reliable data [5].

In Ethiopia, BC is a significant health concern, with increasing cases reported yearly. The diagnosis and treatment of BC can lead to significant physical, social, and psychological distress. These challenges persist during the post-treatment and recovery phases. The psychosocial impact on BC patients is often tied to the importance of breasts in a woman’s self-image, including aspects of femininity, sexuality, and motherhood [7]. Other concerns include changes in physical appearance and disfigurement post-treatment, fear of cancer recurrence, periods of anxiety and depression, difficulty in maintaining hope, fear of death, and loss of self-esteem [8], affecting both patients and their loved ones, especially partners [9]. Research increasingly underscores the profound effects of body image disturbances among BC survivors, particularly after mastectomy or reconstructive surgery [10]. As Sebri and Pravettoni [11] highlight, tailored psychological interventions addressing body image are essential to improve emotional well-being, as disfigurement can result in long-term psychological distress, compromised self-esteem, and challenges in resuming intimate relationships. The shock of a cancer diagnosis and worries about the future or treatment side effects, such as nausea and fatigue, also pose challenges [12]. Practical issues related to treatment, such as costs and travel, can be burdensome. While relatively uncommon in high-income countries, BC in younger women presents additional challenges due to a more aggressive biology and poorer prognosis [13]. Access to BC screenings, diagnosis, and treatment is scarce throughout Ethiopia, notably in rural areas. Furthermore, even when there are screenings, they are very limited with little to no access to mammograms, leaving self-breast exams as the only option. Consistent with other African studies, a 2021 study in Ethiopia found that women who lived in rural areas were four times more likely to get BC [14]. Teshome et al. [15] found that women with BC seeking treatment at Black Lion Specialized Hospital in Addis Ababa encountered barriers, including accessibility and affordability, while long wait times and fear of side effects affected decision-making.

Other pathways for cancer treatment adherence and decision-making have been through support groups. Support groups for cancer patients, particularly those designed for BC survivors, have demonstrated substantial benefits in improving psychological well-being and quality of life. Research from Alshamsi et al. [16] highlights the positive effects of an 8-week psychoeducational support group for BC survivors in Saudi Arabia. The participants reported reduced anxiety and depression, and enhanced quality of life after engaging in structured group sessions that focused on emotional and mental health. Similarly, Chou et al. [17] found that support groups in Asia, integrating psychoeducation, health education, and stress management, successfully alleviated psychological distress in breast cancer patients. Ussher et al. [18] discovered that the sense of belonging and inclusivity within peer support groups was more crucial to participants than the professional competence of group leaders. This theme is echoed in Stevinson et al. [19], who noted that professional and peer-led groups offered similar benefits to cancer survivors in the UK, with peer-led groups being more accessible. These peer support systems help patients become more involved in their care and provide emotional and social support that can be lacking in other relationships.

With cancer being one of the leading causes of death globally and BC being the most common cancer among women, this study explores decision-making around seeking treatment and assessing the impact of a BC support group in Harar, Ethiopia. Overall, the research suggests that cancer support groups, whether peer-led or professionally facilitated, effectively address survivors’ emotional and psychological needs, enhance their quality of life, and foster a sense of community and belonging. In Ethiopia, the establishment and effectiveness of these support groups are influenced by cultural, social, and economic factors [20]. This research is particularly relevant given that the preponderance of literature surrounding cancer, specifically BC, is generated from one hospital in Addis Ababa with very different social determinants. Furthermore, the study’s emphasis on the importance of support groups highlights the need for comprehensive care that addresses BC patients’ medical, emotional, and social needs. In the context of Harar, Ethiopia, we hypothesize that participation in breast cancer support groups positively influences treatment-seeking behaviors and enhances emotional well-being among survivors, despite the moderating effects of cultural, social, and economic factors. Additionally, the findings will have important implications for policy and program efforts, given the lack of integrating psychosocial programs into cancer treatment currently.

## Methods

### Study area and period

Hiwot Fana Specialized University Hospital, in Harar, serves as a primary healthcare provider and teaching institution in eastern Ethiopia, offering a broad range of services across 46 specialized units. Among these is the Ali Birra Memorial Cancer Center, the only specialized facility in eastern Ethiopia where all newly diagnosed cancer cases are referred for comprehensive management. Officially named on October 11, 2023, in honor of Ali Mohammad Birra, a celebrated Ethiopian musician and advocate for cancer awareness, the center provides advanced oncology services to the region’s population, integrating medical treatment with psychosocial support, peer groups, and community outreach. Data collection for this study took place between May and June of 2024, with transcription, translation, and analysis lasting from June to September 2024.

### Design and procedures

This community case study was carried out in Harar, Ethiopia, using semi-structured, in-depth interviews and focus group discussions to gather data on breast cancer (BC) psychosocial support groups and decisions around seeking treatment. This community case study employed a qualitative design to examine BC psychosocial support groups and the sociocultural dynamics influencing treatment-seeking behaviors in Harar, Ethiopia. The research design was situated within a constructivist research paradigm, which assumes that knowledge and meaning are co-constructed through social interactions and are inherently shaped by context, language, and culture [21]. Aligned with this worldview, a social constructionist framework [22] was used to explore the perspectives and experiences of individuals who either attended the BC support groups or are peripherally associated with them. 

In collaboration with the Hiwot Fana Cancer Treatment Center, one of only three radiotherapy cancer treatment centers in Ethiopia, this study collected data from women attending the BC support groups and key informants, including spiritual leaders, caregivers, and providers in this region. Ethical approval was obtained from the University of Notre Dame (protocol # 24–03–8458) Institutional Review Board on April 1, 2024, and Haramaya University (#116/2024) Institutional Health Ethics Review Committee (IHERC) ethical committee on May 20, 2024.

### Participants

Eligibility criteria for the FGDs included those who attended the BC psychosocial support groups held at the Hiwot Fana Cancer Treatment Center, individuals 18 or over with a diagnosis of breast cancer, and those who agreed to informed written consent. Key informants were recruited based on their proximity to decision-making among those with breast cancer and included healthcare workers, caregivers of breast cancer patients, and religious leaders such as traditional healers or priests. Sampling was purposive; our aim within the in-depth interviews was to capture diverse stakeholders (e.g., different healthcare professionals, education levels, experiences with breast cancer, and survivorship) to capture a broad spectrum of perspectives on support groups.

The healthcare practitioners at the Hiwot Fana Cancer Treatment Center recruited participants for the FGDs through phone calls. Those who agreed to participate in the FGDs were compensated for transportation to the cancer center, where the FGDs were held. Additionally, key informants were contacted using various methods, including word of mouth and snowball sampling. Researchers met key informants at their preferred locations for each interview, ranging from clinics to the cancer center to the participant’s business or home.

### Data collection

Key informant in-depth interviews were conducted face-to-face using a semi-structured guide in the participant’s native language (Afan Oromo, Amharic) or English. Questions explored where individuals typically seek health and medical information, the influence of cultural and social norms on women’s healthcare decisions, preferred practices or remedies for BC and their integration with modern medicine, potential improvements in BC treatment, and the perceived impact of support groups on cancer patients’ well-being. Probing techniques were used to elicit more in-depth insights into participants’ experiences and perceptions of support groups and lasted 25 to 30 min on average. Comprehensive field notes were recorded immediately following each interview to support reflexivity and assessing data saturation—the point at which sufficient, nuanced, and intricate data had been gathered to fully address the research question and determine whether sampling was concluded [23]. All interviews were audio-recorded, then translated and transcribed by individuals fluent in the participant’s native language and English (for interviews not conducted in English) using either Otter.AI or a Microsoft Word document for final transcriptions.

Focus group discussions took place at the Hiwot Fana Cancer Treatment Center, including the traditional coffee ceremony that typically precedes their monthly psychosocial support group. Questions within the FGDs included how women in the community learn about BC treatment options, including trusted sources, and the immediate concerns upon diagnosis. Additionally, questions were asked about how BC treatment affects relationships with family and community, decision-making dynamics within households, and the family’s or community’s views on attending support groups. Facilitators spoke in the participants’ native language, Afan Oromo, and discussions lasted approximately 60–70 min. The FGDs were audio-recorded and subsequently translated and transcribed by an individual fluent in Afan Oromo and English using a Microsoft Word document. All data derived from key informant interviews and focus group discussions were stored in a University of Notre Dame Google Suite drive, with only researchers conducting data collection and analysis having primary access.

### Data analysis

Data were analyzed using a reflexive thematic analysis following Braun and Clarke’s [24] methodological guidelines, employing a predominantly inductive, data-driven approach due to the limited understanding of support group experiences in Ethiopia. Two researchers, one responsible for translating and transcribing non-English interviews and FGDs, independently conducted iterative readings of the transcripts to identify recurring patterns. These codes informed the development of thematic constructs encapsulating the salient views of individuals within the interviews and FGDs. To strengthen trustworthiness, the study adhered to Lincoln and Guba’s [25] criteria for credibility, transferability, dependability, and confirmability. Triangulation was achieved by collecting data from multiple sources—patients, caregivers, health workers, and religious leaders—across both individual and group settings, allowing for convergence and comparison of perspectives [26]. Thick description was used in reporting to provide rich, contextual accounts that support transferability to similar settings [27]. An audit trail was maintained to document analytical decisions, ensuring dependability and allowing for external review of the research process [28]. The lead researchers also engaged in reflexive journaling throughout the study to critically examine their positionality and its influence on data interpretation.

## Results

This study included a total of 21 female participants in the focus group discussions. Additionally, 14 participants took part in in-depth interviews with a range of key informants to provide comprehensive insights. These interviews included three traditional healers, one religious healer or priest, five family members (two daughters and three sisters of participants of the support group), one coworker of a support group member, one general practitioner, and three surgical residents currently in specialty training. This approach allowed for a diverse range of perspectives, capturing insights from both the women directly affected by the breast cancer support group and the broader community and professional network involved in their care.

## Sociodemographic characteristics of focus group discussion participants 

The participants in this study represent a range of ages, reflecting the diversity in the experiences and support needs of women diagnosed with breast cancer who engage in support groups. The age of participants in the FGDs ranged from 30 to 70 years (Table 1). Among the 21 women in the FGDs, nine were 30–39 years, nine were between 40 and 49, and three were between 50 and 70. The majority of participants identified as Christian Orthodox (14), followed by Muslim (5) and Protestant (3). While 12 participants were from Harar, the remaining participants came from other regions. Specifically, one participant was from Chiro, which is approximately 200 km away from the center where the support group and the FGD were conducted. All participants used public transportation as a means to travel to the support group, the furthest coming from Chiro, paying 540 Ethiopian Birr per month to attend. The time since diagnosis varied among participants, with most being diagnosed within the past few years. Six of the women had been diagnosed within the past year, ranging from 8 months to just under 1 year. Another seven women had been diagnosed 1 to 3 years ago. The remaining seven participants had been diagnosed more than 3 years ago, with one participant’s diagnosis being 20 years ago.

**Table 1:** Sociodemographic characteristics of focus group discussion participants

	Total (*n* = 21)	
	*N*	Percentage %
Age		
30–35	5	23.80%
36–40	6	28.50%
41–45	4	19%
46–50	3	14.30%
51–55	1	4.80%
56–60	0	0%
61–65	1	4.80%
66–70	1	4.80%
Religion		
Protestant	3	14.30%
Orthodox Christian	13	61.90%
Muslim	5	23.80%
Kebele		
Alemaya	1	4.80%
Aweday	1	4.80%
Boreda	1	4.80%
Chiro	1	4.80%
Dire Dawa	5	23.80%
Harar	11	52.20%
Jigjiga	1	4.80%
Time since diagnosis (years)		
< 1 year	2	9.50%
1	4	19%
2	6	28.60%
3	5	23.80%
4	1	4.80%
5	1	4.80%
> 5 years	2	9.50%
Family history of cancer		
Yes	3	14.30%
No	18	85.70%
Married		
Yes	16	76.20%
No	5	23.80%
Number of children		
0	3	14.30%
1	2	9.50%
2	7	33.40%
3	5	23.80%
4	2	9.50%
5 or more	2	9.50%
Cancer treatment received		
Completed	4	19%
Hormone therapy	12	57.10%
Chemotherapy	1	4.80%
Radiation	2	9.50%
Multiple treatments	2	9.50%
Time in the support group (months)		
1–5	5	23.80%
6–10	11	52.40%
> 10	5	23.80%
Support group attendance		
Regular	19	90.50%
Irregular	2	9.50%

The majority of participants (18) reported no family history of cancer. However, three women indicated a family history of cancer, including one with a grandmother who had cancer, one with a history of esophageal cancer, and one with a history of intestinal cancer. Seven participants reported being married for more than 10 years, with durations ranging from 11 to 32 years. The number of children among the women ranged from zero to six. The treatment plans of the participants varied, with many undergoing hormonal therapy at the time of this study. The time attending the BC support group varied as well, with durations ranging from 4 to 12 months. And 17 participants attended the support group regularly, while two participants attended irregularly.

### Focus group discussions

Thematic analysis revealed eight key themes and several subsequent sub-themes shaping participants’ experiences in breast cancer support groups (Table 2). These included Influence *from* Community and/or Family, the Effects of Sharing within Survivorship, Influence *on* Community and/or Family, Family Challenges and Lack of Support, Pre-existing Support System, Financial Barriers, Logistical Issues, and Knowledge about Breast Cancer.

**Table 2:** Focus group discussion themes and sub-themes

Theme	Sub-themes
Influence from Community and/or Family	Traditional Medicine
	Healthcare Professionals
Effects of Sharing within Survivorship	Shared Fear of Death
	Hope & Motivation
	Creating Interpersonal Connection
Influence on Community and/or Family	-
Family Challenges & Lack of Support	-
Pre-existing Support System	-
Financial Barriers	Treatment & Therapy
	Access & Transportation
Logistical Issues	Growing Pains
Knowledge about Breast Cancer	Considered Taboo
	Perceptions of Alternative Treatments
	Limited Awareness of Modern Medicine

#### Influence from community and/or family

This theme refers to how social networks, including family and community, affect a person’s treatment decisions for breast cancer. Community and family networks were key influencers in treatment decisions, often shaping the initial choice of care. Traditional Medicine was captured as one of the sub-themes wherein this was considered informal practices and beliefs related to health and healing that are passed down through generations. Traditional remedies, rooted in cultural practices, were often the first course of action. One participant remarked, “Most of the society advocates for traditional medicine, especially in rural areas.” Another Respondent exclaimed, “In my area, many people trust traditional healers more than doctors because they lack exposure to modern medicine.” While traditional medicine was a starting point for many, healthcare professionals played a critical role in shifting perceptions toward modern treatment. Healthcare Professionals were noted as another sub-theme and were defined as individuals who are trained in modern medical practices and play a vital role in the diagnosis, treatment, and management of various health conditions. One participant noted, “I did have people who are healthcare professionals who convinced me to seek care. They also helped me understand more about medical treatment.”

#### Effects of sharing within survivorship

This theme refers to the impact of discussing personal experiences in a support group setting, where individuals connect through shared emotional and psychological challenges. The support group environment emerged as a transformative space where participants found space, hope, and a sense of community by sharing their experiences. This theme includes three sub-themes, Shared Fear of Death, Hope & Motivation, and Creating Interpersonal Connection.

Shared Fear of Death was the widespread fear of mortality experienced by breast cancer patients after receiving their diagnosis. This fear often encompasses concerns about the uncertainty of the prognosis, the potential for cancer recurrence, and the impact of treatment, which can lead to heightened anxiety about the future. Women expressed profound worries about their futures and their families. One participant shared, “After I heard I had cancer, I was so anxious because, at that time, I had no children of my own. I thought I was going to die.” Another stated, “I was mostly afraid of death and leaving my children alone. It was the hardest thing to think about.”

While fear was certainly present, Hope & Motivation radiated positive emotional and psychological benefits derived from participating in a support group, where individuals facing breast cancer can find encouragement and strength. Through the experiences shared by fellow survivors, members may gain hope as they witness the resilience and recovery of others, which can inspire optimism for their future. One woman exclaimed, “I started to hope for the future. I hope to do so many things in the future.” With others sharing similar feelings.

Creating Interpersonal Connection was the last sub-theme and was defined as the social benefits that individuals gain from participating in a support group, such as the formation of meaningful relationships and a sense of belonging. Through the shared experience of breast cancer, members may develop strong feelings of camaraderie, often considering the group a “second family.” These interpersonal connections are built on mutual understanding and shared struggles, allowing individuals to relate to peers who truly understand their journey.

#### Influence on community and/or family

This theme describes the impact that breast cancer survivors have on their social networks following treatment, where they may serve as change agents by advocating for early detection, screening, and modern treatment approaches. Many participants became advocates in their communities, breaking taboos and encouraging others to seek early detection and treatment. One participant shared, “Through the process of seeing me get better, people in my community started believing that someone with cancer could survive.”

#### Family challenges and lack of support

There were several difficulties faced within family dynamics, including limited encouragement or understanding of medical needs. One woman describes her situation stating, “Nobody was supportive of me having the breast surgery operation that I was going to have my breast removed.” She goes on to say that after she had her mastectomy, “After they saw them remove the breast, they cried.” Others described similar situations where they were not supported in their decision to seek medical treatment.

#### Pre-existing support system

While some women described the lack of support, others characterized their support systems as the availability of emotional, social, or practical support, such as family, friends, or community resources, for patients undergoing breast cancer treatment. One woman reflected, “My husband was with me throughout the entire process. He tried so hard not to express his emotions in front of me, but I know he was very afraid.”

#### Financial barriers

Financial obstacles that impact access to necessary treatment, therapy, or transportation for healthcare services were considered Financial Barriers. Economic challenges were a pervasive issue for participants, impacting their ability to access timely care and treatment. Two sub-themes emerged from this theme, Treatment & Therapy and Access & Transportation. Many participants cited the high cost of chemotherapy and surgery as a significant barrier. One participant shared, “The expense of chemotherapy and surgery was overwhelming. I had to seek help from family and friends to afford it.” Likewise, Access & Transportation brought challenges related to the affordability and availability of transport to healthcare facilities (from nearby and far distances). A participant explained, “Traveling to the city for treatment was so expensive and exhausting. Many women in my area can’t afford it, so they don’t even try.”

#### Knowledge about breast cancer

Participants expressed the need for awareness and understanding of breast cancer diagnosis, treatment, and survivorship within the community. Three sub-themes were identified: Taboo, Perceptions of Alternative Treatments, and Limited Awareness of Modern Medicine.

Breast cancer was considered taboo due to cultural or social stigma that discourages open discussion or acknowledgment of breast cancer. One woman stated, “In my community, breast cancer is seen as a death sentence, and people don’t even talk about it.” Furthermore, participants noted Perceptions of Alternative Treatments were views on non-modern medicine methods (ex. traditional/religious remedies). Several women talked about using both alternative treatments concurrently with modern medicine. A participant described how she “would also use the holy water first. I need to seek medical treatment and know my current situation.” However, others spoke of women who used alternative options exclusively, “There was a person in my community who refused breast removal surgery. She tried alternative treatments, but her condition worsened, and eventually, she passed away.” Limited Awareness of Modern Medicine contributed to the lack of knowledge regarding contemporary medical treatments and their effectiveness; specifically stating, “I still think there is a problem in creating awareness, modern treatment, educating the community, and such things as people trusting more traditional medicine.”

#### Logistical issues

Participants discussed issues with scheduling, the size of the support groups, and whether splitting the support groups into smaller ones was beneficial or not. Organizational challenges within the support groups were evident, particularly in terms of scheduling and group dynamics. Additionally, as the groups grew in size, maintaining meaningful and intimate discussions became increasingly challenging—as indicated in the sub-theme Growing Pains. The growing number of members made it harder to sustain the level of interaction that was initially possible in smaller groups. In describing the changing group dynamics, one member stated that she “did not like that things have been divided into two [support] groups.”

### Key informant interviews

Key informant interviews included people with a range of different experiences with cancer, ranging from caregivers to spiritual leaders to providers. Thematic analysis was used to analyze the 14 interviews, resulting in nine themes with most having subsequent subthemes (Table 3). Final themes include Awareness Creation, External Pressure, Influence of Cultural and Religious Beliefs, Trust in Alternative Medicine, Healthcare System Challenges, Cycle of Death, Perceived Understanding of Cancer, Emotional Psychological Impact of Breast Cancer, and Attitudes Toward Psychosocial Support Groups.

**Table 3:** Key informant themes and sub-themes

Theme	Sub-themes
Awareness Creation	Lack of Information
	Leveraging Social Media
	Mis/Disinformation
External Pressure	Community/Family Impact
	Spousal Decision-Making Power
Influence of Cultural and Religious Beliefs	Stigma toward Mastectomy
	Autonomy of the Patient
Trust in Alternative Medicine	Affordability & availability
	Convenience
Healthcare System Challenges	Early Screening
	Delay in receiving care
	Limited treatment options
Cycle of Death	Transition to Modern Treatments from Alternative
	Simultaneous use of Modern & Alternative Treatments
Perceived Understanding of Cancer	Perception of Cancer as a Death Sentence
	Lack of Knowledge Around Causes of Breast Cancer
Emotional Psychological Impact of Breast Cancer	No Subthemes
Attitudes Towards Psychosocial Support Groups	Effect on participants
	Impact on communities

#### Awareness creation

Awareness Creation was defined as a potential solution in addressing modern forms of treatment through educating and informing communities and individuals about health conditions and treatments to improve health-seeking behaviors. Three sub-themes were identified within this theme: Lack of Information, Leveraging Social Media, and Mis/Disinformation. One participant stated, “[there is a] need to create awareness about the illness and disease, not only breast cancer but also…other cancers. Awareness needs to be created.”

The first sub-theme included Lack of Information, which included significant challenges with compliance due to misinformation or lack of comprehensive information. The next, Leveraging Social Media, refers to the use of social media platforms to spread health information and influence behavior, which is largely employed by healthcare professionals to reach communities. The final subtheme, Mis/Disinformation, represents the spreading of false and/or misleading information that may negatively impact decision-making. Interviewees shared that “a few years back, cancer was seen as a death sentence, and the advocacy that was made regarding cancer was that it was deadly and nobody survives…because of that, people are very fearful of cancer” and that “we can address the urban area with…social media”.

#### External pressure

External pressure was identified as pressure that arises from sources other than the individual, in this case women, that ranges from active persuasion to passive information sharing or misinformation. Two sub-themes emerged: Community/Family Impact as well as Spousal Decision-Making Power.

Community/Family Impact emphasizes the influence of the broader community and family in shaping a woman’s health-related decisions, while Spousal Decision-Making Power refers to the degree to which a husband decides whether a woman seeks medical treatment within the Ethiopian context. According to one interviewee, “Usually sick people don’t make the decisions for themselves…most of all the people around them matter in making the decisions.” Alongside this, it was shared that the “husband, the mom, and the neighbors [influence] the health-seeking decision of the womens” and that because the “husband is the owner of the finances…[and] to seek medical attention [the woman] needs financial [help]. So…the owner [of financial power] is the husband.”

#### Influence of cultural and religious beliefs

The Influence of Cultural and Religious Beliefs was defined as the impact of community-wide cultural norms and religious practices on health that could affect medical treatment. As one individual shared, “Because of the cultural influence, especially in this part of our community where the majority of people are Muslims, they don’t want to expose themselves especially when related to private parts.” Sub-themes include Stigma toward Mastectomy and Autonomy of the Patient.

Stigma toward Mastectomy highlights negative perceptions within the community about mastectomy that lead to hesitancy in pursuing this treatment. The Autonomy of the Patient focuses on beliefs that can limit a woman’s ability to choose what she wants, especially as family approval is often required or sought for health decisions. Participants shared that people believe “they will die after mastectomy. They will die after six months to one year” and that “women lack the autonomy to make decisions to seek treatment because of financial dependency, economic dependency [on] men, [and] women are usually sidetracked by the influence of other women.”

#### Trust in alternative medicine

Alternative medicines are those considered in lieu of modern medicine. Trust in Alternative Medicine is the degree of choice to use other ways of treatment due to cultural beliefs or ease. This theme is derived from people sharing that “since [their] community is traditional, they go initially to…traditional healers before [modern medicine]”. This theme encompassed two sub-themes: Affordability and Availability and Convenience.

Affordability and Availability underscore the perception that alternative medicines are often seen as more affordable and accessible than modern medicine. Convenience refers to the perceived ease of use among alternative treatments in contrast to modern treatments. A respondent explained that “many cancer patients cannot afford the treatment and the economic burden [making] some people choose…and seek traditional medicine”. Another shared that “while working, [she] could easily access the traditional medicine and it’s easier for [her] to take this traditional medicine because of that”.

#### Healthcare system challenges

Healthcare System Challenges are issues with the healthcare infrastructure that make effective screening, diagnosis, treatment, and care difficult to address. There are three sub-themes: Early Screening, Delay in Receiving Care, and Limited Treatment Options.

Early Screening highlights the lack of accessible screening options, often leading to late-stage diagnoses. Delay in Receiving Care points to challenges in accessing care due to distance and financial constraints. Limited Treatment Options describe insufficient access to necessary drugs and medical equipment, limiting treatment effectiveness and efficacy. Interviewees described that, “early detection of the disease is low because in our country also there is no guideline to screening for diseases” and that “sometimes the drugs are not available, and [they] have to pay extra every 21 days.”

#### Cycle of death

The Cycle of Death is a pattern perceived by the community wherein those who are diagnosed with cancer will soon die after seeking modern medical treatment. This perception is a result of delaying modern treatments, preferring alternative modes of treatment that often lead to worsening conditions. Finally, at the point when a person returns for modern treatment, they are often presented with an advanced stage of cancer where they will be put on a palliative care plan. As one participant described, “[some] women will not come to us because of shame [so] she [will] repeat the cycle as it was. If they go to have their breasts removed, then they attribute the death not to the cancer but to the surgery where she died. She died because she had her breast removed. A lot of…misconceptions.” Two sub-themes supporting this theme are: Transition to Modern Treatments from Alternative and Simultaneous use of Modern & Alternative Treatments.

Transition to Modern Treatments from Alternative is defined as the shift from using alternative medicine initially just after diagnosis and then reverting to modern medicine after an advanced stage. Meanwhile, Simultaneous use of Modern & Alternative Treatments describes patients blending modern and alternative treatments, with mixed outcomes. One participant recounted “They come to [modern medicine] after they try most traditional medicines even [if the cancer] has reached another complicated level”, and another said, “Breast cancer treatment is the integration of traditional and modern medicines. Take remedies from both sides and use it together.”.

#### Perceived understanding of cancer

The Perceived Understanding of Cancer refers to community and individual perceptions and misunderstandings about cancer that influence how they approach treatment. Sub-themes include Perception of Cancer as a Death Sentence and Lack of Knowledge Around Causes of Breast Cancer.

The first sub-theme, Perception of Cancer as a Death Sentence, references the belief that a cancer diagnosis will lead to death, leading to fear and reluctance to seek modern treatment. The second, Lack of Knowledge Around Causes of Breast Cancer, describes misconceptions around the causes of BC leading to ineffective prevention and treatment practices. Interviewees shared that “cancer was seen as a death sentence” and that “the cause[s] for breast cancer [include] weather conditions, extreme hot and extreme cold, eating more sweats, drinking more water, and sunlight.” A traditional healer said breast cancer resulted from “breastfeeding, abnormality, menstruation…caffeine and soft drinks.”

#### Emotional psychological impact of breast cancer

The Emotional Psychological Impact of Breast Cancer theme represents the personal and emotional struggle of breast cancer on patients, including denial around the prognosis and stigma related to potential treatment options, specifically mastectomy or chemotherapy. Hearing from respondents that “cancer by itself makes patients agitated and anxious” and that a patient “always feels sad about having the mastectomy and she hasn’t told almost anybody” reflected this theme.

#### Attitudes towards psychosocial support groups

The final theme identified was Attitudes toward Psychosocial Support Groups, and it describes community and patient perspectives on the value and impact of psychosocial support groups. This theme comprises two sub-themes, Effect on Participants and Impact on Communities.

Effect on Participants is the perceived benefits of support groups for participants through sharing experiences, resources, and relationship-building. Impact on Communities is the role of support groups in advocacy and their influence on community attitudes toward breast cancer. As one interview described “When [her relative] comes back home from psychosocial support group meetings, she smiles more, you [can] see happiness from her face” and another said that her loved one “now has full confidence to talk about cancer in the community…trying to convince others to have surgery”.

## Discussion

This study explored the impact of breast cancer (BC) support groups on the survivorship and treatment decision-making of women in Harar, Ethiopia, a region with limited access to cancer care resources. The findings highlight the central role of psychosocial support in improving cancer survivors’ emotional and psychological well-being, as well as how cultural, social, and economic factors influence treatment decisions. This discussion integrates the study’s findings with relevant literature to emphasize the broader implications for cancer survivorship in sub-Saharan Africa (SSA) and offers recommendations for future research and practice.

The cycle of death—where patients seek medical care only after cancer has reached an advanced stage—remains a significant challenge in low- and middle-income countries (LMICs), including Ethiopia. Delays in diagnosis and treatment are compounded by cultural perceptions, logistical barriers, and poor healthcare infrastructure [29]. Henke et al. [30] explore how delayed cancer presentation is a widespread issue across LMICs, emphasizing that when patients seek treatment only after the disease has advanced, the chances of survival decrease dramatically. This delay leads to a pattern of palliative care being the most feasible option, perpetuating a vicious cycle of late-stage cancer diagnoses and poor prognosis. From the community’s perspective, including participants in this study, it also perpetuates the idea that medical care leads to or causes death.

Breaking this cycle requires comprehensive strategies to improve early detection and reduce the delays in seeking treatment. Early screening for breast cancer, when implemented alongside public awareness campaigns, can increase the detection of cancer at stages where treatment is more effective [31]. Unfortunately, the lack of resources and awareness in regions like Ethiopia exacerbates the problem, where screening services are scarce, and patients often rely on traditional medicine before turning to modern healthcare services [32]. These delays in seeking medical care, particularly in rural and underserved areas, significantly undermine the effectiveness of modern treatment options, reinforcing the need for earlier intervention [30].

Awareness creation is integral to overcoming the cycle of death in cancer care. The findings of this study assert the critical need for community-based education about breast cancer, its risk factors, and the importance of timely medical care. Vale et al. [32] emphasize that educational initiatives, particularly those tailored to low-resource settings, can reduce the stigma surrounding cancer and increase the uptake of screening and treatment. Zahid et al. [33] underscore the importance of health-seeking behavior education in the context of oral cancer patients, citing that individuals from lower socioeconomic backgrounds often delay seeking medical treatment due to a lack of awareness about the seriousness of the disease and cultural beliefs about cancer. Consequently, leveraging social media and community outreach programs to educate populations about the signs and symptoms of BC can facilitate earlier detection and treatment, as evidenced by recent studies on public health interventions in similar settings [29]. In addition to raising awareness about cancer, these initiatives can help challenge cultural misconceptions surrounding cancer treatment. Similarly, Henke et al. [30] highlight how education is essential in changing societal perceptions, particularly regarding traditional versus modern medicine. When patients are educated about the benefits of early medical intervention and the risks of delaying treatment, they are more likely to seek care sooner, improving survival rates and reducing the incidence of late-stage diagnoses.

A significant finding from the focus group discussions (FGDs) and key informant interviews is the overwhelming influence of family and community on treatment decisions. In Ethiopian culture, the role of husbands and family members in decision-making is paramount, with many women reporting that their treatment choices were influenced by the wishes or opinions of their families. This mirrors the findings of McCutchan et al. [31], who note that external pressures, including the influence of family and community members, can either encourage or hinder a woman’s decision to pursue cancer treatment. Particularly in rural areas, where traditional values are strongly upheld, spousal decision-making power often dictates the course of medical intervention [32].

In addition to family influences, traditional healers also play a significant role in adjudicating cancer treatment in many African communities. Traditional healers are often seen as the first point of contact for health issues, including cancer, and their role as trusted health advisors is deeply embedded in cultural practices. They are viewed not just as caregivers but also as spiritual and cultural leaders who provide both physical treatments and emotional support. This cultural authority can significantly delay or hinder cancer patients from seeking timely medical care, as many individuals in rural settings are more likely to trust these healers over modern doctors [31]. This dynamic is evident in other studies as well, where patients are reluctant to pursue modern medical interventions due to the strong influence of traditional beliefs and the role of traditional healers in shaping health behaviors [34–36]. The reliance on traditional healers for cancer care highlights the complex interplay between modern medicine and traditional healing practices, which can sometimes create barriers to early diagnosis and treatment. As Brand et al. [29] explain, fear of diagnosis and the belief that cancer is incurable often lead individuals to delay seeking medical treatment, opting instead for traditional remedies. Such attitudes, when combined with the power dynamics within families, exacerbate delays in diagnosis and treatment, reinforcing the cycle of death in cancer care.

The study also underscores the significant financial barriers and healthcare infrastructure challenges that impede access to timely cancer care. Participants reported that the high costs of treatment, as well as the financial burden of traveling long distances to receive care, were major obstacles to seeking treatment. Al-Sukhun et al. [37] argue that the price-efficacy dilemma in cancer care—where the cost of treatment far outweighs the perceived benefit in low-resource settings—often leads to decisions to forgo or delay treatment. In Ethiopia, where access to radiotherapy, chemotherapy, and screening services is limited, patients often resort to alternative medicine due to its lower cost and greater availability [30]. Moreover, the lack of a robust healthcare infrastructure exacerbates these financial barriers. The high cost of medical bills coupled with the duration and cost of public transportation have been identified as significant barriers to timely cancer care in both Asian and African studies [38–40]. In several studies conducted in various African countries [41–43], patients with breast cancer relied heavily on social networks, including relatives, community members, and churches, to alleviate the financial burden of medical expenses and transportation. This support system was instrumental in reducing the pretreatment interval by enabling more timely access to care.

Finally, breast cancer support groups can play a crucial role in addressing the significant barriers posed by stigma, which often impedes individuals from seeking timely medical care. Many cancer survivors report that their participation in these support groups helped them cope with the fear of recurrence and death by providing a sense of hope and motivation. These findings align with research by Gottlieb and Wachala [43], who emphasize that support groups offer a space for individuals to feel less isolated in their struggles, gain insights into different ways of managing their situations and emotions, and experience comfort through the group dynamic. For cancer patients, particularly those without strong external support systems, the emotional benefits of peer support are invaluable in counteracting the pervasive fear of diagnosis and death. The sense of hope generated within these groups is particularly significant in communities where traditional family or community networks may not always provide adequate emotional or psychological support.

Furthermore, support groups can help combat the cultural stigma surrounding cancer, which has been consistently identified as a significant barrier to help-seeking behavior. In many LMIC settings, including parts of Asia and Africa, cancer is associated with shame, secrecy, and fear of social rejection, particularly in the case of breast cancer [34, 44]. This stigma often delays individuals from seeking care, as they associate a cancer diagnosis with an inevitable death. Support groups can offer a counterbalance to these cultural norms by providing a safe space where participants can openly discuss their diagnoses, treatment options, and experiences, reducing the sense of isolation that cancer patients often feel [31]. By fostering an environment of mutual support and understanding, these groups challenge the negative perceptions of cancer and promote a more positive, proactive approach to treatment, helping patients overcome the social stigma and fear that may otherwise prevent them from seeking timely medical attention.

This study provides valuable insights into the role of breast cancer support groups in eastern Ethiopia, a region with limited prior research on this topic. A key strength lies in its comprehensive approach, which captures diverse perspectives from participants, including survivors, healthcare providers, caregivers, and spiritual leaders. This inclusivity enriches the understanding of the multifaceted influences on treatment decision-making and survivorship. However, given the unique regional dynamics, the findings may not be generalizable beyond the specific cultural and social context of Harar, Ethiopia. Additionally, the risk of information being lost in translation during data collection and analysis may have impacted the depth and accuracy of specific themes despite efforts to ensure linguistic and cultural fidelity. Likewise, although trusted facilitators were used to foster openness and minimize response bias, the study relied entirely on self-reported data from breast cancer survivors and key informants, which may be subject to recall bias, social desirability bias, and subjective interpretation. These limitations suggest the need for further research across different settings and with larger, more diverse populations and mixed-methods research.

This study’s findings suggest that support groups could be a valuable resource not only for breast cancer patients but also for individuals dealing with other chronic health conditions, such as cervical cancer. Given the financial and infrastructure limitations in Ethiopia and other LMICs, future research should explore the potential for scaling support groups to other forms of cancer care and incorporating support mechanisms for caregivers of cancer patients and providers. Additionally, further studies should investigate the long-term impacts of support groups on cancer survivors’ psychosocial and clinical outcomes.

## Supplementary Information

Below is the link to the electronic supplementary material.

Supplementary file1 (PDF 42 KB)

Supplementary file2 (PDF 39 KB)

## Data Availability

The datasets generated during and analyzed during the current study are available from the corresponding author upon reasonable request.
