# Correction to: Exploring the impact of breast cancer support groups on survivorship and treatment decision‑making in Eastern Ethiopia: a qualitative study

**DOI:** 10.1007/s00520-025-09538-y

**Published:** 2025-05-16

**Authors:** Nahom G. Belete, Meera Bhakta, Tara Wilfong, Mahlet Shewangizaw, Edilawit Abebaw Abera, Yehenaw Tenaw, Michael Shawel, Habtamu Seife, Biruk Habtamu, Nahom Wondwossen, Elizabeth A. Wood

**Affiliations:** 1 Hiwot Fana Specialized University Hospital, Harar, Ethiopia; 2 College of Science, University of Notre Dame, Notre Dame, IN USA; 3 School of Public Health, College of Health and Medical Sciences, Haramaya University, Harar, Ethiopia; 4 Eck Institute for Global Health, University of Notre Dame, Notre Dame, IN USA

**Correction to: Supportive Care in Cancer (2025) 33:419**

**https://doi.org/10.1007/s00520-025–09475-w**

The correct names of the authors are Yehenaw Tenaw and Edilawit Abebaw Abera, rather than Yehanaw Tenaw and Edil Abebaw.

The original article has been corrected.

