# Supplementary material for: Exploring the impact of breast cancer support groups on survivorship and treatment decision-making in eastern Ethiopia: a qualitative study
Source: Support Care Cancer. 2025 Apr 26;33(5):419. doi: 10.1007/s00520-025-09475-w (PMC12031865; doi:10.1007/s00520-025-09475-w)
Supplement: Supplementary file 1 — Supplementary file1 (PDF 42 KB) [file 520_2025_9475_MOESM1_ESM.pdf]

Focus Groups:

1. How do women in your community learn more about treatment options for breast cancer?
    a. Are there specific sources/people that are trusted more?
2. When you found out you had breast cancer, what were some of your immediate concerns? (examples: your job, family life, treatment, etc.)
    a. What, if any, were some of the barriers to receiving care?
3. How has the breast cancer psychosocial support group affected your overall well-being?
4. What have been some of the benefits and drawbacks of the breast cancer psychosocial support group?
5. Has it influenced how you speak about cancer and cancer treatment, in general, within your community?
6. Has your experience receiving breast cancer treatment affected your relationship with your family, friends, or community?
7. How are decisions about healthcare made in your household?
    a. How does the opinion of your family/community affect your decision to seek treatment?
8. How does your family/husband or community feel about you going to the support group?
