# Supplementary material for: Exploring the impact of breast cancer support groups on survivorship and treatment decision-making in eastern Ethiopia: a qualitative study
Source: Support Care Cancer. 2025 Apr 26;33(5):419. doi: 10.1007/s00520-025-09475-w (PMC12031865; doi:10.1007/s00520-025-09475-w)
Supplement: Supplementary file 2 — Supplementary file2 (PDF 39 KB) [file 520_2025_9475_MOESM2_ESM.pdf]

In-Depth Interviews:

1. Where do you typically seek information about health and medical treatments?
2. How do cultural and social expectations shape women's healthcare decisions in your community?
3. Are there specific practices or remedies preferred for breast cancer?
    a. How are they managed with modern medicine?
4. What changes could improve breast cancer treatment?
5. How do you think support groups impact someone with cancer’s well-being?
